# Pathogenic Bacteria of the Human Mouth

**Published:** 1888-06

**Authors:** W. D. Miller

**Affiliations:** Berlin, Germany


### Independent Practitioner.

VOL. IX. JUNE, 1888. NO. 6.

NOTE.—No paper published or to be published in another journal will be accepted for this department. All papers must be in the hands of the Editor before the first day of the month preceding that in which they are expected to appear. Extra copies will be furnished to each contributor of an accepted original article, and reprints, in pamphlet form, may be had at the cost of the paper, press-work and binding, if ordered when the manuscript is forwarded. The Editor and Publishers are not responsible for the opinions expressed by contributors. The journal is issued promptly, on the first day of each month.

### Original Communications.

### PATHOGENIC BACTERIA OF THE HUMAN MOUTH.

### INTRODUCTION.

BY PROF. W. D. MILLER, BERLIN, GERMANY.

In point of temperature, culture-material, moisture, etc., the fluids and accumulations of food in the human mouth present the best possible universal medium for the development of bacteria, both pathogenic and non-pathogenic. We should accordingly expect that among the many different kinds of bacteria that are continually entering the oral cavity, either with the food or air, pathogenic kinds may be included, and that these may proliferate for a certain length of time, or may even establish themselves as permanent occupants of the mouth. Many facts point to the truth of the statement that pathogenic bacteria may be present in the mouth without manifesting themselves in any other way than the ordinary parasites of the oral cavity, as long as the mucous membrane remains intact. If, however, the resistance of the soft parts has been impaired by any constitutional cause (scorbutus, syphilis, etc.), a *locus minoris resistentiæ* will thereby be created, at which the bacteria may manifest their specific action in local inflammation, suppuration, etc. Or if the continuity of the soft parts has been destroyed, as in tooth extraction, wounds of the mucous membrane, etc., an entrance into the blood or lymph vessels is thereby provided, which may lead to still more serious results (abscess, pyæmia, septicæmia, etc.).

In this manner, I am convinced, many of the diseases of the gums and contiguous parts (not excepting pyorrhœa alveolaris, as I shall endeavor to prove later), as well as the majority of the infections following tooth extractions, are to be accounted for.

It is only within the last few years that dentists and physicians are beginning to understand the importance of the human mouth as an incubator for bacteria, and the disastrous results which may follow the neglect to keep the mouth in a proper condition, placed as it is at the entrance of the digestive and respiratory tracts, communicating with so many contiguous cavities, and so often the seat of minor surgical operations which permit of infection.

Every tooth extraction which is not performed under antiseptic precautions is nothing less than an inoculation, and whether the subject proves refractory or not will depend upon a variety of circumstances, as the size of the wound, the resistance of the parts, the character and number of bacteria entering the wound, etc.

Parré (*Dental Record,* October, '87) reports a case of chronic pyæmia which originated in a diseased wisdom-tooth. Poncet (*Gazette des Hôpitaux,* No. 19) describes a case of osteitis, which, originating in a carious tooth, led to a general septic infection, terminating fatally in forty-eight hours. Tripp (*Dental Record,* August, '87) observed a case of inflammation of the brain following an alveolar abscess. Ritter (*Deutsche Monatsschrift für Zahnheilkunde,* August, '87,) a case of septic blood poisoning from a carious tooth, terminating fatally, and Mosetig-Moorhof (*Oest.-Ung-Vierteljahrsschrift,* January, '87), a case of fatal osteomyelitis following extraction.

Dental literature furnishes a large number of cases in which death, owing to infection, has occurred from one to ten days after extraction of a tooth. Naturally, however, not all cases of infection have so severe a character, in very many cases remaining localized, giving rise only to a slight swelling or suppuration of the parts, or by no means unfrequently to severe purulent inflammation, osteitis, necrosis, etc. Such cases must be familiar to every one ; in fact, where resection of the alveolus is practiced, extraction of a root in an unclean mouth without the observation of antiseptic precautions will, in nine cases out of ten, be followed by more or less inflammatory reaction of a septic nature.

The wounding of a finger by an instrument just used in a dirty human mouth may prove a very serious matter, as I can testify from personal experience, having been confined to my room for two weeks by an exceedingly painful lymphangeitis and phlegmon of the hand, contracted in this manner. Scratching the finger on a sharp root in an unclean mouth has produced much more serious results.

Many years ago Leyden and Jaffé pointed out that the bacteria which are found in the mouth, even in a state of perfect health, may, under predisposing circumstances, give rise to severe lung diseases; also James Israel, who has given much time to the study of the transportation of bacteria from carious teeth, describes a number of cases of abscesses on the neck, chronic pyæmia, etc., in which he was able to find the primary source of infection in the mouth. The oral cavity, when it is allowed to become the seat of extensive parasitic vegetation, may also have a most deleterious action on the general health, leading to concomitant affections of so severe a nature that the primary affection may be, and very often is, entirely overlooked by the physician.

A series of interesting communications dealing with this question were made by Von Kaczorowsky (*Deutsche Med. Wochenschrift*, 1885, 33-35).

After several years of practical experience and a long series of observations, Von K. came to the conclusion that many cases of loss of appetite, nausea, dyspepsia and consequent general ill-health, were to be attributed to an unclean condition of the oral cavity, independently of the condition of the teeth themselves. Acting upon this conclusion he was able to treat such cases with marked success by simply insisting upon repeated cleansing and sterilization of the mouth and throat.

"When we take into consideration that affections of the mucous membrane of the mouth may *per continuum et contiguum* spread over the whole mouth and throat, and thence upwards and downwards over the respiratory and digestive tracts, through the Eustachian tube to the internal ear and brain, from the nose through the lachrymal canals to the eye, through the sieve of the ethmoid to the membranes of the brain; furthermore, when we remember that the irritation of the terminal branches of the quintus in the gums may produce irradiations in other branches of this nerve or irritations in near and distant organs of the body—" * when we keep all these things in mind, and remember at the same time that many people, as Von K. rightly says, carry about constantly an amount of filth in the mouth which they would not tolerate for a moment upon the skin, then we can readily account for many troubles which otherwise appear inexplicable and stubbornly resist every treatment by the usual internal remedies.

Individuals whose teeth are in a state of almost complete ruin, whose gums are swollen and suppurating, whose breath testifies to the intense fermentation (in some cases putrefaction) going on in their uncleaned mouths, receive one prescription after another, or are sent from one bathing place to another to no purpose whatever, when a thorough cleansing and disinfection of the mouth, accompanied by the necessary dental work, would restore them to health in a very short time.

Up to the present time very few attempts have been made, in cases of severe infection through the oral bacteria, to cultivate the specific bacterium by which the infection was brought about, and indeed a very grave hindrance is opposed to the study of the bacteria and bacteritic affections of the mouth, in the circumstance that such a large number of different kinds is to be found, and that many of the oral bacteria are not to be cultivated on any of the artificial media at present in use, among others leptothrix buccalis, vibrio buccalis (spirillum sputigenum) and spirochæte denticola, and further a thick spirillum and a leptothrix which occurs in short, stiff, pointed threads. These are found in every mouth, and notwithstanding the fact that thousands of attempts have been made to cultivate them, no one has ever yet succeeded. We must, therefore, constantly bear in mind in the study of infectious diseases of the mouth (we might say in the study of all infectious diseases), that possibly the specific bacterium in the case under consideration is one which cannot be cultivated with the means now at our disposal.

---

* Von Kaczorowski, Deutsche Med. Wochens., 1885, page 570.

(TO BE CONTINUED.)

